# ARMA Model for Tracking Accelerated Corrosion Damage in a Steel Beam

**DOI:** 10.3390/s25082384

**Published:** 2025-04-09

**Authors:** Sina Zolfagharysaravi, Denis Bogomolov, Camilla Bahia Larocca, Federica Zonzini, Lorenzo Mistral Peppi, Marco Lovecchio, Luca De Marchi, Alessandro Marzani

**Affiliations:** 1 Department of Civil, Chemical, Environmental, and Materials Engineering—DICAM, University of Bologna, Viale Risorgimento2, 40136 Bologna, Italy; denis.bogomolov2@unibo.it (D.B.); alessandro.marzani@unibo.it (A.M.); 2 Advance Research Center on Electronic Systems “Ercole De Castro” (ARCES), University of Bologna, Viale Carlo Pepoli, 3/2, 40123 Bologna, Italy; camilla.bahialarocc2@unibo.it (C.B.L.); federica.zonzini@unibo.it (F.Z.); lorenzomistral.pepp2@unibo.it (L.M.P.); l.demarchi@unibo.it (L.D.M.); 3 Department of Electrical, Electronic and Information Engineering—DEI, University of Bologna, Viale Risorgimento 2, 40136 Bologna, Italy; 4 Department of Computer Science, Bioengineering, Robotics and Systems Engineering—DIBRIS, University of Genova, Viale Causa, 13, 16145 Genova, Italy; 7647356@studenti.unige.it

**Keywords:** ARMA model, accelerated corrosion, damage evolution detection, structural health monitoring

## Abstract

This paper proposes an enhanced vibration-based damage detection index leveraging autoregressive moving average (ARMA) time-series modeling. The method relies on the fact that material deterioration alters the vibration features of the structure. Thus, the proposed method employs an innovative usage of the ARMA time-series modeling to capture subtle shifts in the vibration response. Specifically, first, a reference ARMA model is fitted on the acceleration response of the undamaged structure. Next, a damage index (DI) is built from the goodness of fit between predicted responses from the reference ARMA model and the actual measured damaged-state acceleration data. Experimental validation was conducted on a steel beam subjected to a controlled accelerated corrosion (up to 40% thickness loss), simulating real-world degradation. Accelerations due to quick-release tests were collected using two accelerometers, along with thickness measurements providing ground-truth damage progression. Results demonstrate that the proposed method can provide sufficient sensitivity in detecting early-stage corrosion progression. This finding highlights the proposed usage of ARMA model’s potential for early structural damage detection, offering significant advantages for safety and maintenance strategies in civil engineering applications.

## 1. Introduction

Damage, defined as any deviation from a structure’s initial state that adversely affects its performance, typically exposes through the degradation of material properties, variations in geometry and mass distribution, leading to alterations in the structural response. Early detection of damage is crucial for minimizing future repair costs, ensuring structure longevity and preventing catastrophic failures. One of the types of degradation in steel structures is the corrosion of the material itself. As materials erode or deteriorate over time, the properties of the structure modify w.r.t. to the designed ones, invoking the need for the early diagnostic and monitoring of corrosion.

To this purpose, Structural Health Monitoring (SHM) methods, nowadays in continuous development, are considered. Among several others, vibration-based SHM methods, are well established and accepted for early damage detection. Vibration-based SHM methods can be classified based on various criteria, including their behavioral characteristics (linear or nonlinear), computational approach (physics-based or data-driven), excitation type (forced or ambient), and the analysis domain (time, frequency, or time-frequency domain) [1]. From the viewpoint of analysis domain, vibration-based methods for damage detection can be approached in two primary ways: (i) leveraging time-series modeling techniques, such as autoregressive moving average (ARMA) models, and (ii) extracting frequency domain features, such as natural frequencies and modal shapes. Time-domain methods offer a data-driven perspective by characterizing the statistical properties of measured signals, whereas frequency-domain methods focus on shift in the extracted modal parameters.

Compared to the time-domain methods, the frequency-domain method is considered to be more stable, as the extracted features are independent of the type of excitation. While frequency-domain methods offer more stability, time-domain approaches like ARMA models remain critical for detecting localized or transient damage that may not be exhibited clearly in modal parameters. Their ability to model non-stationary signals and subtle statistical changes makes them particularly suited for early-stage deterioration detection.

One of the most effective time-series modeling categories is the one based on autoregressive models, as their filter bank implementation efficiently approximates the structure’s frequency response function. To this end, different statistical time-series methods are proposed which are compatible with output-only vibration inspection, the most popular of them including the autoregressive (AR), the moving average (MA), and the autoregressive moving average (ARMA) model [2].

Although many researchers have utilized time-series modeling to detect specimen damage, only a few have specifically targeted the application of time-series modeling in early-stage deterioration detection. Monavari et al. [3] proposed a signal-based approach utilizing autoregressive (AR) time-series residuals. A novel AR model order estimation algorithm was established that could enhance the sensitivity of the AR model prediction concerning deterioration. While AR models simplify analysis by focusing on autoregressive components, ARMA models are exploited for their capability to efficiently capture both autoregressive and moving average dependencies in structural response data while maintaining a balance between model complexity and computational cost. This dual capability enhances sensitivity to subtle, nonlinear changes induced by progressive deterioration.

Traditional ARMA time-series modeling for damage detection typically quantifies damage measuring the distance between ARMA models fitted to response of specimen under different damage conditions. In this study, a novel use of ARMA models in autoregressive form for early detection of corrosion damage in a steel specimen is investigated. In the proposed method, a new damage index is defined based on the goodness of fit between the predicted response of a reference ARMA model (fitted to the response of an undamaged specimen) and experimentally measured response. The effectiveness of the proposed damage index will be assessed by comparing it with two conventional methods in the field. Finally, to evaluate the robustness of the proposed method, the Pearson correlation coefficient between the new damage index and applied level of damage will be computed.

To experimentally validate the proposed methodology, an experimental setup for the early detection of corrosion-induced damage in a steel specimen was designed. To achieve a controlled yet time-efficient simulation of corrosion damage, an accelerated corrosion method was employed. This study explores the effectiveness of ARMA models in an autoregressive form for the early detection of corrosion-induced damage in steel specimens subjected to accelerated corrosion.

The paper is organized as follows: Section 2 briefly describes the experimental setup, as well as the selected methodologies, while Section 3 presents the results obtained for three methodologies. Concluding remarks are reported in Section 4.

## 2. Materials and Methods

In this section, first a description of the experimental setup including the details of the specimen, the procedure of applying growing damage using accelerated corrosion, as well as the data acquisition and snap tests are provided. Then, methods for detecting the existence of damage inside the specimen are presented.

### 2.1. Experimental Setup

#### 2.1.1. Specimen Description

The test specimen is a laboratory-scale steel cantilever beam carrying a lumped mass at its free end. The beam has a rectangular cross-section of 60 × 5 mm, a total length of 1000 mm, with a free length of 850 mm after being clamped on an anti-vibration table at one end. At the support, Styrofoam elements were placed between the anti-vibration table and the steel specimen. A lumped mass of 5.6 kg was added to the free end of the beam to reduce its natural frequencies, as well as to enhance the participation of lower vibration modes. To ensure the integrity of the beam-chamber connection during the test, it was important to avoid the formation of plastic hinge close to the support of the beam. Therefore, the added mass was limited to 5.6 kg, to avoid inelastic behavior in the beam, especially during the very initial stage of the test. The weights were firmly secured using a hand screw clamp to ensure stability during testing.

**Figure 1:**
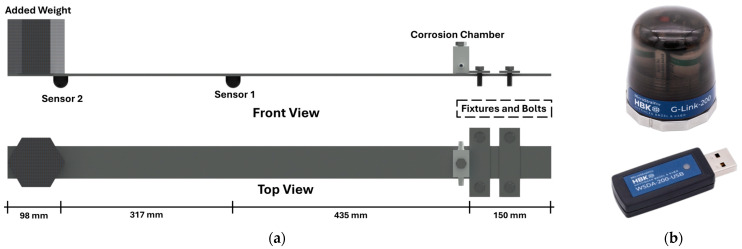
(**a**) Schematic of the test setup, and (**b**) the wireless sensor and the USB key.

Two accelerometers were attached to the bottom side of the beam using double-sided adhesive tape, positioned at 435 mm and 752 mm from the support to capture the dynamic responses of the beam during the test. Each accelerometer has a mass of 122 g. Sensors were positioned close to the location of antinodes of the first two flexural modes, to properly capture the relevant dynamic information. The wireless sensing network consisted of two G-Link-200 triaxial MEMS wireless accelerometers (LORD Sensing MicroStrain, Williston, VT, United States) [4]. The devices are battery-powered and offer an adjustable measurement range of ±8 g with 20-bit resolution. They are capable of transferring data in real-time with a synchronization of ±50 μs. The accelerometers have a low noise density of 25 µg/(√Hz) with ±25 mg zero-g offset level. Figure 1 provides a schematic representation of the specimen, as well as the acceleration sensors used.

The nominal modal characteristics of the specimen were determined from a finite element simulation in OpenSees [5]. To this end, a 3D finite element model was built. Elastic beam column elements with two nodes, each of which having six degrees of freedom, were used to discretize the beam. The beam was originally discretized into ten elements. To accurately incorporate the added masses from the sensors, as well as the additional masses near the free end of the beam, further discretization of the beam was applied in that region. To replicate the inertial effects of both the added mass and accelerometers, nodal masses were assigned at defined locations. Simulation was done incorporating steel material properties (Elastic Modulus, E=200 GPa, and density, $\rho =7850\;kg/m^{3}$). Nodal masses were applied to account for the additional mass from the sensors. Table 1 presents the simulated natural frequencies of the first four modes of vibration.

#### 2.1.2. Continuous Damage Growing

**Figure 2:**
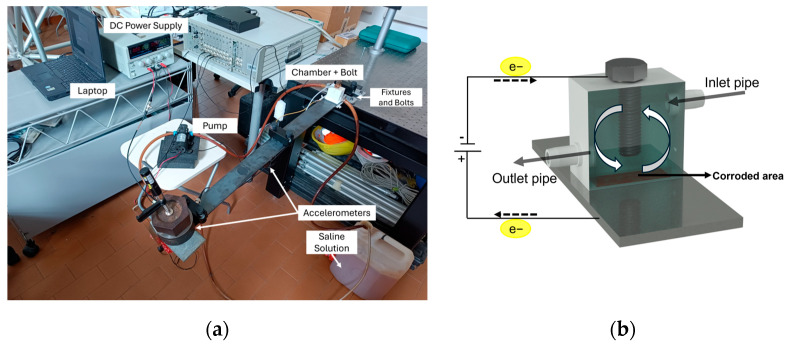
(**a**) Overview of the experimental test setup, and (**b**) schematic of the corrosion chamber and water circulation system.

To induce an accelerated corrosion process on the beam, a dedicated experimental setup was developed. The setup, inspired by the work of Rao et al. [6] and Zou and Cegla [7], is depicted in Figure 2a. In particular, near to the beam support, a reaction chamber, as shown in Figure 2b, was securely sealed to the steel specimen using silicone glue to prevent any possibility of leakage. The upper section of the chamber contains an aperture for a bolt that acts as the electrode, with a 10 mm gap between the bar and the bolt. A potentiostat supplies a constant electric current of 1.1 A between the bolt (cathode) and the bar (anode). The chamber’s lower section remains open to enable interaction between the electrodes and a 3.5 weight percent (w.t%) NaCl solution, simulating typical seawater salt concentration. To reduce the formation of corrosion products that can act as a barrier and affect the metal’s corrosion rate, a DC12V/60W micro diaphragm pump, powered by a DC power supply at 1.7 V, was used to circulate the solution through the chamber at a controlled rate of 0.16 L per minute. This continuous flow decreases the precipitation of corrosion products, especially iron oxide, helping to maintain a constant corrosion rate by inhibiting the oxidation of the iron oxide to insoluble iron hydroxide [6]. Moreover, this flow minimizes temperature fluctuations during the corrosion process, which is crucial since temperature variation can affect the modulus of elasticity of the material, which can further cause changes in the modal characteristics. The rate of the loss of the thickness (mm/h), referred to as Corrosion Rate (CR) can be estimated by the following formula obtained from Faraday’s law of electrolysis [6]:

(1) $$CR=-\frac{{3.6\times 10^{6}M}_{Fe}I}{2FA_{c}\rho_{Fe}}$$

where $M_{Fe}$ is steel molar mass (0.055845 mol/kg), I is the applied current (A),  F is the Faraday’s constant (96,485.3329), $A_{c}$ is the area of the plate under corrosion, and $\rho_{Fe}$ is the steel material density (7850 kg/m^3^). Considering the applied current of 1.1 A, and the internal area of the used chamber as 40 × 20 mm, CR corresponds to 0.18 mm/h.

To quantify corrosion-induced thickness loss and reconstruct the corrosion profile, beam thickness was measured periodically using a portable Digital Ultrasonic Thickness Gauge TN80-0.1US, with a resolution of 100 μm (Sauter GmbH, Balingen, Germany). The beam’s corroded backside was divided into five zones (P1–P5), and the measurement interval was 30 min, except for the last two measurements, spaced one hour apart. In total, the experimental procedure extended over a duration of 8.5 h.

Table 2 shows the measured thickness over the five defined zones with time. Based on the measurements, the rate of corrosion at the initial stage of the experiment (80 min) was 0.165 mm/h. As the test continued, the rate was reduced and the average rate of corrosion for the whole test was 0.11 mm/h. Considering the fact that the rate of corrosion may decrease due to the dissolution of iron oxide in saline electrolytes and the formation of protective corrosion products on the substrate surface [8], the measured rate shows a significant correlation with the theoretical rate of 0.18 mm/h.

**Figure 3:**
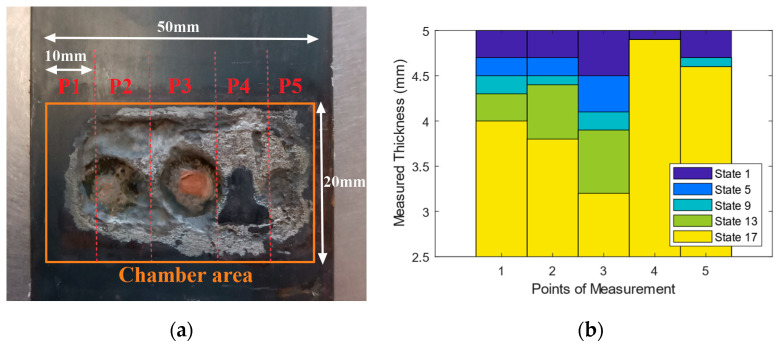
(**a**) Corroded specimen, and (**b**) the thickness profile of the beam section at different states.

Figure 3a shows a photo of the real corroded area after the test. As can be seen, the corrosion was not uniform over the beam width. This could be caused due to the gluing of the chamber to the specimen. During the test, the saline water was circulating to keep the temperature low; however, in some cases, the temperature could increase due to a non-proper circulation of water, which causes the melting of the glue over the corrosion surface. Another reason could be the existence of rust or other byproducts forming a protective layer over the surface of corrosion, or on the bolt (cathode) that prevents the uniformity of the corrosion. Based on the observed values, the profile thickness at different time of test has been plotted in Figure 3b.

#### 2.1.3. Free Vibration Tests

Snap tests (quick-release displacement) were conducted intermittently to evaluate damage progression. These tests involved applying a low-amplitude displacement at the beam’s free end to send the beam to free vibration state. The dynamic response was recorded via triaxial accelerometers (sampling frequency: 256 Hz, acquisition window: 120 s). The acquisition window length was determined to ensure the imposed displacement from snap tests was fully dissipated, assuming a 0.5% damping for the first mode of vibration.

### 2.2. Data Processing

#### 2.2.1. Data Pre-Processing

Acceleration data were pre-processed before being fed to further steps. First, each acceleration time history was de-trended by removing the best straight-line fit. After, the de-trended data were filtered using a second-order Butterworth filter with corner frequencies of 0.15 Hz and 75 Hz to remove noise effects. The second-order Butterworth filter was selected due to its flat frequency response in the passband, helping to preserve the signals characteristics in the frequency range of interest. The lower and upper cutoff frequencies were selected in such a way as to make sure that the filter did not attenuate frequencies within the range of interest, spanning from 1.65 Hz (i.e., 1st flexural mode) up to 55 Hz (3rd flexural mode).

#### 2.2.2. General Overview of Modal Parameter Extraction

**Figure 4:**
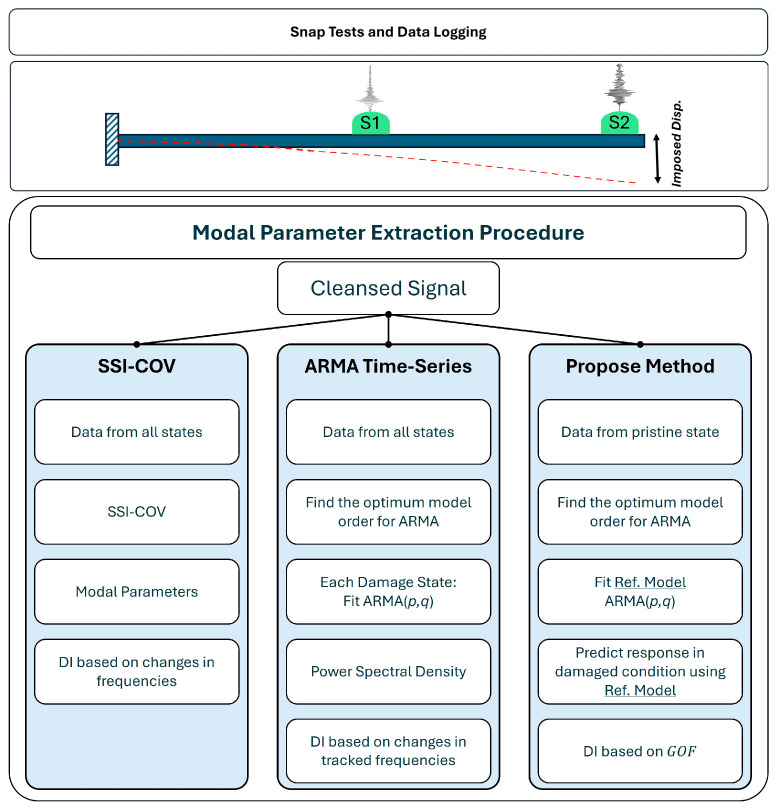
Data gathering and analysis structure. Snap test includes imposing an initial displacement to the specimen which is shown by a red dashed line. Accelerometers are shown with two green spheres attached to the top surface of the beam.

The modal properties of beam are identified with the aid of three different methodologies: (I) the Covariance-Based Stochastic Subspace Identification (SSI-COV) algorithm, taken as reference modal analysis method and used for comparison; (II) a conventional ARMA model for time-series modeling; and (III) the proposed ARMA model regression, based on the goodness-of-fit metric, as explained in the following sections and illustrated in Figure 4. In the next subsections, a summary of the methods is explained. For more detailed information about the SSI-COV and ARMA time-series modeling, readers are encouraged to refer to references [9,10,11,12].

I. Covariance-Based Stochastic Subspace Identification

The Covariance-Based Stochastic Subspace Identification (SSI-COV) method operates within the time domain and relies on the covariance of output-only measurements, making it ideal for scenarios where input data are either unavailable or difficult to measure. This method estimates the state–space model by organizing vibration data into a block Hankel matrix, a structured matrix that encapsulates lagged time-series data. The Hankel matrix is divided into past and future components: the past block contains historical data used to predict future behavior, while the future block holds the measurements to be predicted. Careful selection of block size is critical. It should be large enough to encapsulate the system’s dynamics without introducing redundancy or amplifying noise.

To form the Henkel matrix, the output measurements are systematically arranged into overlapping blocks such that each block represents a snapshot of the system’s dynamic behavior over a specified time window. Specifically, if the measured outputs are denoted as a time series, the Henkel matrix is formed by stacking rows where each row comprises successive output values offset by a fixed lag. The first block consists of the first set of measurements, the second block shifts one step ahead, and this process is continued until the matrix spans the entire available dataset. This structure ensures that both recent and old data are used to capture the evolution of the system’s dynamics.

The identification process involves projecting future measurements onto past ones. The resulting projection matrix is then factorized into an observability matrix and a state sequence using singular value decomposition (SVD). The system’s order is determined by the number of significant (non-zero) singular values. By incrementally shifting the division between the past and future measurements, the alternative projection, observability, and state sequence matrices are generated. From these matrices, the system’s properties can be calculated through an overdetermined set of linear equations. Although the specifics of the methodology are not detailed here, further information can be found in reference works [13].

In the SSI-COV method, model reduction is performed by selecting the ‘n’ largest singular values for estimating the system matrix ‘A’, where ‘n’ represents the system order. However, due to measurement noise and modeling inaccuracies in real-world applications, determining the appropriate system order—i.e., differentiating between significant and negligible singular values—can be challenging, particularly when attempting to automate the identification process. To address this, a stabilization diagram approach is implemented. Stabilization diagrams illustrate the evolution of modal parameters, such as natural frequencies, damping ratios, and mode shapes, across varying system orders. Physical modes are identified as those with consistent modal parameters across different systems orders.

At each system order, the identified modes are compared with those from the previous order. A mode is considered ‘stable’ if the relative change in natural frequency is less than 1%, the relative change in damping ratio is less than 50%, and the Modal Assurance Criterion (MAC) between the current and previous mode shapes exceeds 0.9. Modes that remain stable across three consecutive orders are classified as physical modes of the structure. Furthermore, any vibration mode with a damping ratio outside the range of (0–15)% are classified as spurious mode. The optimal system order is determined by selecting the lowest order that captures the maximum number of stable physical modes. These stability criteria, specifically the error tolerances of 1% for natural frequencies and 50% for damping ratios, are based on empirical data, engineering judgment, and prior experience. The identified modal frequencies can be used to form a damage index. In the current study, the damage index (*DI*) is defined to track the evolution of damage into the specimen as follows:

(2) $$DI=100\times \frac{1}{k}\sum_{i=1}^{k}\frac{\left|\omega_{i}-\omega_{baseline}\right|}{\omega_{baseline}}$$

where k is the number of modes of interest, and $\omega_{i}$ and $\omega_{baseline}$ are the modal frequency of interest and the baseline modal frequency, respectively.

II. Conventional ARMA Time-Series Model

An autoregressive moving average process (ARMA) can be used to describe the dynamic response of an oscillating structure and to obtain its spectral parameters by means of a parametric procedure. An ARMA process is given by [14]:

(3) $$x_{n}=-\sum_{i=1}^{p}a_{i}x_{n-i}+\sum_{i=0}^{q}b_{i}e_{n-i}$$

where $a_{i}$ and $b_{i}$ are the autoregressive (AR) and moving average (MA) coefficients, respectively; p and q are the model orders of the AR and MA process, respectively; and $e_{n}$ is a zero-mean white noise process that is supposed to excite the structure.

The general behavior of the ARMA model is dependent on the selection of the *p* and *q* orders. When selecting the model order for an ARMA model, it should be low enough to ensure simplicity and generalizability across different datasets while being high enough to minimize residuals and accurately capture the system’s dynamics. In other words, an overly simple model increases residual errors, whereas a higher-order model may overfit and fail to generalize to other datasets [3]. Several techniques are available to evaluate the selection of ARMA model orders, such as information-based criteria (Akaike Information Criteria (AIC) and Bayesian Information Criteria (BIC)), root mean square error (RMSE), and autocorrelation and partial autocorrelation functions of model residuals.

**Figure 5:**
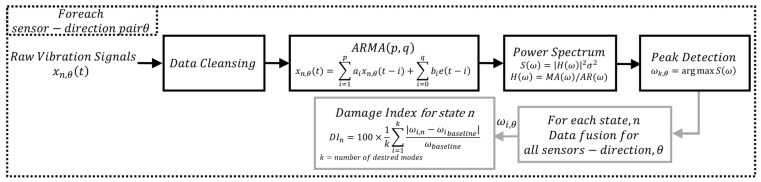
Sample schematic of conventional use of ARMA process for damage detection.

The conventional application of ARMA time-series modeling involves using them as a time-series fitting tool through the identification process. The suitability of ARMA models as effective processing algorithms for frequency characterization lies in their implicit implementation of a filtering process, where the frequency response function corresponds to a proxy for the structural dynamics. In this regard, the ARMA models are used as a fitting tool to each set of data, as illustrated in Figure 5. An ARMA model is fitted to the signal for each consecutive damage condition, generating a dataset of parameters that encapsulates the full progression of structural conditions, ranging from the pristine state to fully developed damage condition. The variation in the model parameters reflects variations in critical modal parameters of the specimen, including shifts in the natural frequencies. To track the variation in the dominant frequencies of the specimen, after fitting an ARMA model for each damage state, an automated procedure was employed to track the dominant frequency across different states. A tolerance was introduced to the procedure representing the acceptable range of frequency variation for a single mode between the two consecutive damage states. To this end, the power spectral density of the ARMA model is calculated and the peaks are detected and tracked to be used as a damage index. The power spectral density of the ARMA process is calculated as follows:

(4) $$PSD_{ARMA}=\sigma^{2}\cdot |H{\left(\omega \right)|}^{2}$$

where σ is the variance of the input noise, which is estimated from the output data during model fitting, and $H\left(\omega \right)$ is the transfer function of the ARMA model as follows:

(5) $$H\left(\omega \right)=\frac{MA(\omega )}{AR(\omega )}=\frac{1+\sum_{k=1}^{p}b_{k}z^{-k}}{\sum_{k=0}^{q}a_{k}z^{-k}}$$

where the $MA\left(\omega \right)$ and AR(ω) are the MA and AR polynomials, respectively, and $\omega =e^{\frac{i2\pi f}{f_{s}}}$. The corresponding frequencies to the peaks of the $PSD_{ARMA}$ are extracted as the natural frequencies of the specimen, and shall be used for defining a damage index, equal to Equation (2) in the previous section.

Theoretically, an ARMA(*p*,*q*) process with the order of p equal to *n* and q equal to *p* − 1 would be sufficient to model the responses of structures excited by truly random Gaussian distributed input motion when there is no noise in the measurements. However, the presence of noise in the measurements in real-world cases makes it impossible to estimate the orders of p and q a priori [15], specifically in the case of the automatic identification process. Although there are no unique best parameters for the ARMA model, by exploring various possible orders for the AR and MA components using the autocorrelation function (ACF) and partial autocorrelation function (PACF), and then evaluating them with an information criterion such as AIC, the optimal model orders can be selected as target configuration [16].

III. Damage detection based on ARMA model regression and the new Damage Index (DI)

**Figure 6:**
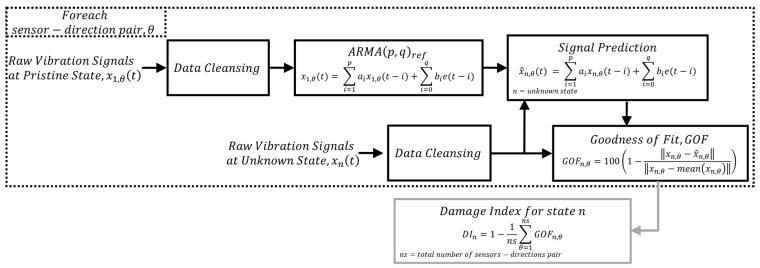
Schematic of use of ARMA model in autoregressive form for damage detection.

As an alternative to standard spectral-oriented applications of ARMA processes, the novel method presented in this work explores its usage in the native autoregressive form for detecting the existence of damage. In this method, which is described in Figure 6, a reference ARMA model is fitted to the vibration response of the structure in its pristine state under a free vibration motion to encapsulate the dynamic behavior of the undamaged structure. When the structure is subjected to varying damage conditions, the set of parameters obtained in the reference state is applied to predict the vibration response which is compared with the measured system response under these new conditions. The goodness of fit between the predicted and the measured response serves as a damage sensitive index.

Since the model parameters are strictly linked to the vibration pattern of the structure, when a shift in the modal response occurs as a consequence of structural degradation (e.g., corrosion), a variation of the filter pole locations is expected. Those variations cannot be properly captured by the reference ARMA model, as they were fitted based on a different dataset and reflect another spectral pattern. This procedure is applied sensor-wise and for each sensing direction (θ). Then, the arithmetic means for goodness of fit from all the sensor-location combinations is used to build the damage index for a given damage state, and is expressed as follows:

(6) $$DI=1-\frac{1}{ns}\sum_{\theta =1}^{ns}GOF_{n,\theta }$$

where *ns* is the total number of combinations for sensors and directions. The $GOF_{n,\theta }$ for the datasets are calculated as follows:

(7) $$GOF_{n,\theta }=100\left(1-\frac{\left\|x_{n,\theta }-{\hat{x}}_{n,\theta }\right\|}{\left\|x_{n,\theta }-mean\left(x_{n,\theta }\right)\right\|}\right)$$

where $x_{n,\theta }$ and $\hat{x_{n,\theta }}$ are the measured and predicted signal at sensing direction θ at state n.

## 3. Analysis and Results

### 3.1. SSI-COV Results

Analysis started by finding the model order based on the stabilization diagram using the cleansed data from the undamaged condition dataset. The analysis here is carried out in MATLAB R2023b using the OoMA Toolbox [17]. The stability analysis was performed across model orders ranging from 2 to 40. The green points refer to stable modes, which satisfy the stability criterion described before. The horizontal line shows the final selected order of the state–space model. The logarithm of averaged power spectral density of all acceleration channels is superimposed on the stabilization diagram and shown on the right axis.

**Figure 7:**
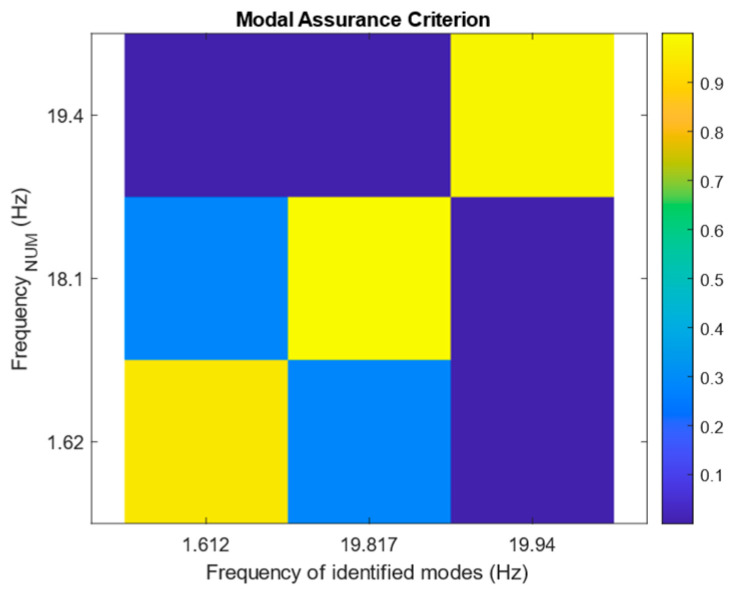
MAC values between the identified mode shapes of the pristine state and the numerical mode shapes.

As can be seen, the stable frequencies range around the first and second flexural modes, as well as the first transversal mode. Spurious modes also appeared around the first transversal mode frequencies. These modes probably resulted from the rotation of the modes due to the type of excitation. During the analysis of the pristine dataset, the spurious poles are removed from the analysis using the MAC values between the identified modes and the numerical mode shapes; however, for the other datasets, the obtained mode shapes from the pristine state are used as a reference for analysis. Figure 7 shows the MAC value graph, comparing the identified mode shapes of the pristine state with those of numerical analysis.

**Figure 8:**
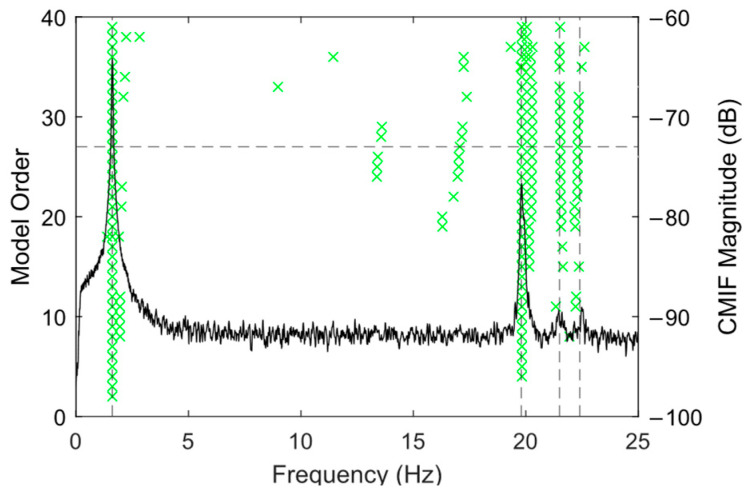
Stabilization diagram using the dataset for undamaged condition. Green x symbols show the identified frequencies at each model order. Black dashed line shows the location of peaks in the frequency response.

Figure 8 depicts the stabilization diagram using the pristine state data. As depicted by the dashed black line, the analysis concluded that a system order of *n* = 27 was the lowest order to include all stable modes within the frequency band of interest.

**Figure 9:**
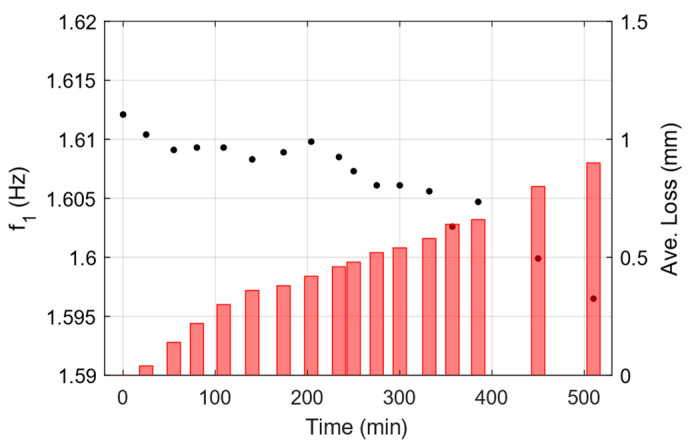
Identified frequency of first mode at each damage state using SSI-COV method (Black dots, left axis). Measured thickness reduction is shown on the right axis using red bars.

Following the analysis, identified natural frequencies of the system are extracted and reported in. There is reasonable agreement between the first three identified natural frequencies and those calculated numerically. The third natural mode did not appear in the analysis, as it might not get excited by the type of excitation which is done by applying an initial displacement to the free end of the beam. Figure 9 depicts the identified frequency from the SSI-COV method for the first flexural vibration mode. The identified frequency shows a descending trend, which is in good agreement with the stiffness reduction assumption following the applied corrosion (i.e., thickness loss) close to the support of the beam.

### 3.2. Conventional ARMA Process for Deterioration Detection

In this approach, as a conventional method, an ARMA model is directly fitted to the channel-wise vertical acceleration data collected from two sensors attached to the specimen and for different damage states. The predicted responses from each ARMA model are subsequently processed to monitor variations in the frequency of the first two flexural vibration modes of the specimen.

To find the optimum model orders (p,q) for the ARMA model, the ACF and PACF of data for the pristine state were used. Based on the results obtained, the investigation range of p and q were selected to be from 9 to 40 and 35 to 45, respectively. A grid search was done to find the model with the lowest AIC value and highest *GOF* in time domain. As a result of the grid search, the top five models and their corresponding AIC and *GOF* values for the two sensors (in vertical directions) are calculated and reported in Table 3. The reason behind the reduced *GOF* for sensor 1 is the lower amplitude of induced acceleration, therefore, the level of noise masks damped acceleration response after a few cycles. Based on the observations, a different set of p and q were selected for each sensor; ARMA(26,36) and ARMA(38,40) models were configured for the sensor in the middle of the beam and for the sensor at the free end of the beam, respectively.

**Figure 10:**
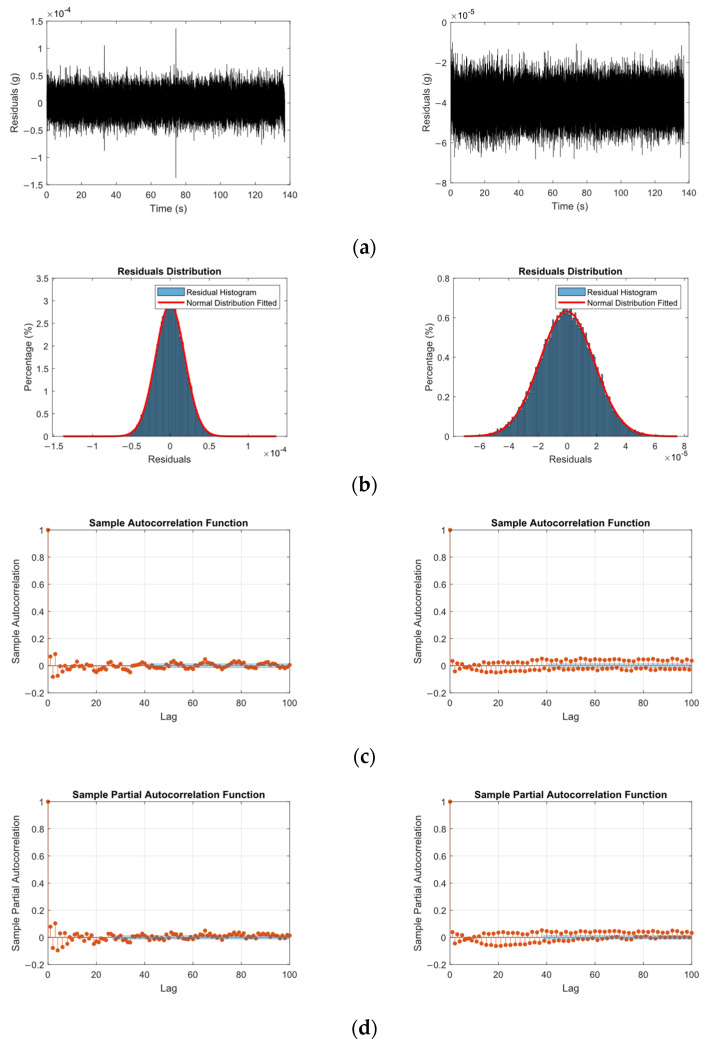
(**a**) Time history, (**b**) the distribution, (**c**) autocorrelation, and (**d**) partial autocorrelation function of the one-step-ahead prediction error for sensor attached at the center of the beam (**Left**) and at the free end of the beam (**Right**).

To verify the randomness of the errors of the fitted ARMA model, residuals were calculated from the one_step_ahead prediction of the model response. These residuals were then examined using the autocorrelation and partial autocorrelation function (ACF and PACF) to ensure no trends remained uncaptured. Figure 10 shows the time-series plot, along with the histogram, autocorrelation and partial autocorrelation function for the pristine state for both the sensors attached to the beam. The analysis shows that both ACF and PACF values drop sharply and stay within 5%. This indicates that the residuals do not exhibit notable autocorrelation and can be considered effectively white noise. Therefore, the ARMA model has adequately captured the underlying structure of the data.

**Figure 11:**
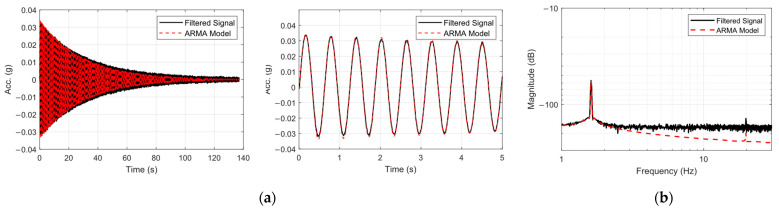
Comparison of the original filtered data and output of the ARMA model in (**a**) time domain, along with zoomed over the first 5 s of response, and (**b**) comparison of the Welch-based frequency-domain spectrum of the measured signal and ARMA model.

The identified frequencies for the first and second natural modes in the pristine state were 1.612 Hz and 19.824 Hz, respectively, which were in agreement with those of numerical analysis. Figure 11 compares the output of ARMA model (fitted to the data) with the filtered measured acceleration response of the free end of the beam in its pristine state, in both time and frequency domains.

At each damage state, and for each sensor–location pair (θ), an ARMA model was fitted. The ARMA model parameters are then used to calculate the PSD, as explained in Section 2.2.2. The peaks of the PSD functions which correspond to the natural frequencies of the specimen are extracted for the first two modes. The identified frequencies are summarized in Table 4, highlighting the variations induced by structural changes.

**Figure 12:**
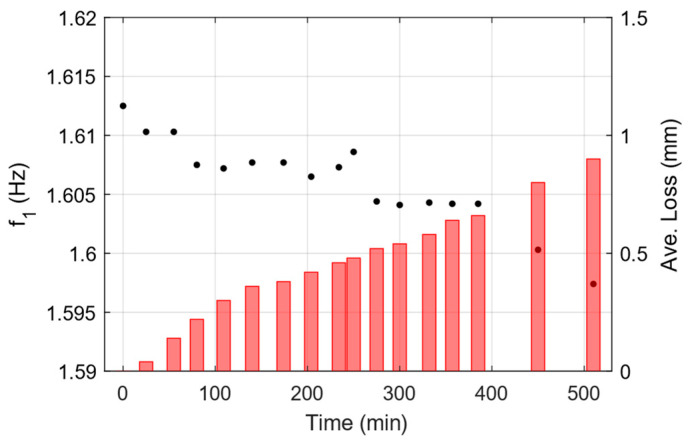
Identified frequency of first mode at each damage state using conv. ARMA modeling (Black dots, left axis). Measured thickness reduction is shown on the right axis using red bars.

Figure 12 shows the identified frequency for mode 1, extracted from the PSD of the ARMA model, fitted to the data from each damaged state. Focusing on only the variation in frequency for mode 1 and the fact that test has been carried out in a controlled environment, changes in the frequency can be linked to the existence of subtle changes into the material. The identified frequencies demonstrate strong consistency with those obtained through the SSI-COV method, and the distinct downward trend in the frequencies confirms the progression of material deterioration.

**Figure 13:**
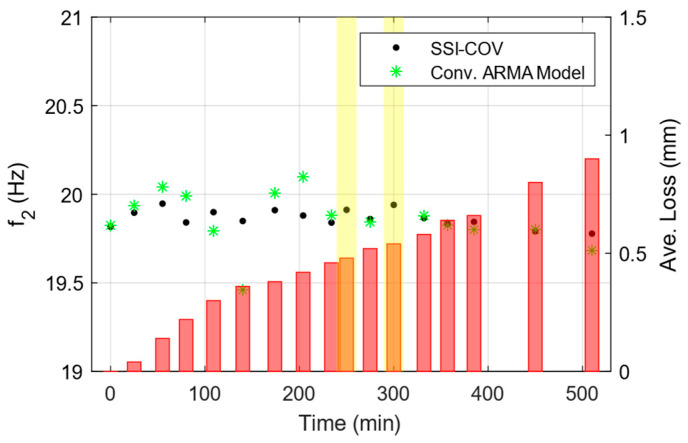
Comparison of identified frequency of second mode at each damage state using two conventional methods (Black dotted and green starred data on the left axis). Measured thickness reduction is shown on the right axis using red bars.

A clear descending trend is observed in the identified frequency of mode 1; however, as illustrated in Figure 13, both conventional methods exhibit fluctuations in the identified frequencies for mode 2. This instability in higher mode frequency poses challenges for developing a reliable damage index chart based on the identified frequencies. Figure 13 presents the identified frequencies for Mode 2 obtained using conventional methods. The two yellow rectangles highlight regions where the conventional ARMA model failed to properly identify the second mode of vibration, since it has not been properly excited in those two states.

**Figure 14:**
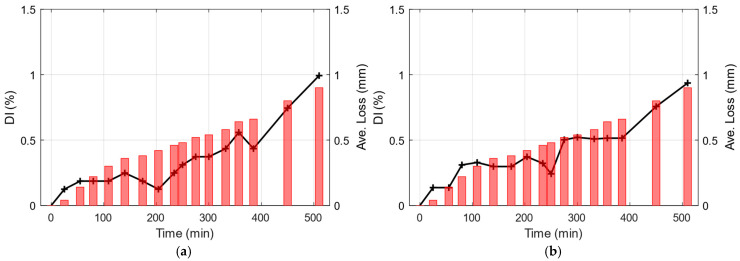
Damage index chart based on (**a**) SSI-COV and (**b**) Conv. ARMA model results (Black solid line with plus symbols). Measured thickness reduction is shown on the right axis using red bars.

In this regard, the damage index charts for the two conventional methods are developed based on the variations observed in the first identified frequency. Figure 14 shows the *DI* chart both for the SSI-COV and Conv. ARMA method. The overall trend of the *DI* chart is consistent with the observed trend in the average thickness loss during the test.

### 3.3. Damage Detection Based on ARMA Model Regression and the New Damage Index (DI)

**Figure 15:**
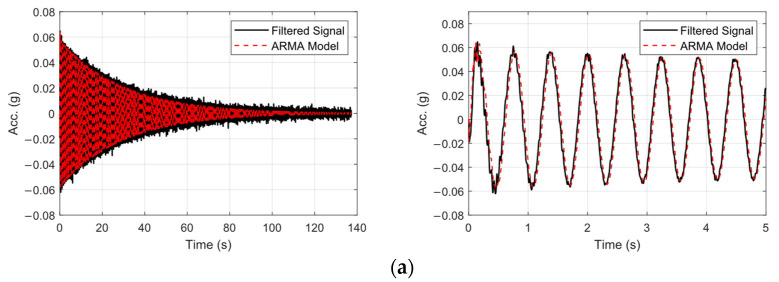

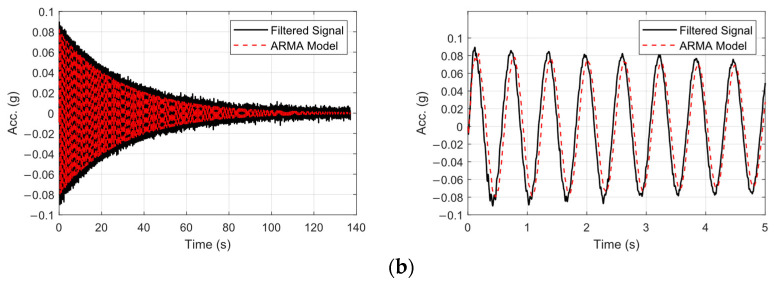
Comparison of the predicted response using Ref. ARMA model and the measured response, (**a**) State 3, and (**b**) State 6 along with their zoomed response over the initial 5 s (**right**).

This section introduces a novel time-series analysis method based on ARMA models. In this method, a reference ARMA model was employed to fit a model to the data from pristine state. The reference ARMA model shall be used in regression form to predict the response of the damaged states. To ensure consistency with prior analyses, the same ARMA model orders are employed for sensor-wise analysis. Within this regression framework, the fitted ARMA model generates a predicted output for each input signal. Input signals corresponding to the pristine state are highlighted with a high value of GOF, since the model is inherently tuned to this baseline condition. Conversely, input signals from damaged states yield to a decreased GOF as a consequence of altered dynamics of the model due to the presence and severity of damage. For each damage state, the GOF of the ARMA model response in the time domain was studied and the damage index for each state was calculated based on the procedure in Section 2.2.2. The same procedure was followed using data for both sensors. As the damage severity increases in time, the ability of the ARMA model to estimate the response of the specimen decreases, which is proof of damage introduced into the specimen which affects the dynamics of the specimen. Figure 15 depicts comparison between the predicted response by the reference ARMA model and the real measured response in damage state 3 (Figure 15a) and state 6 (Figure 15b). As can be seen, as the damage progresses, the ability of the fitted reference ARMA model to predict the response (GOF) is reduced.

**Figure 16:**
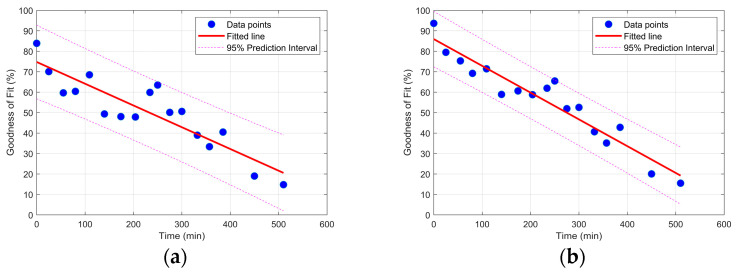
Goodness-of-fit for different damage scenarios for (**a**) acceleration data close to the middle of the beam, and (**b**) acceleration data close to the free end of the beam.

Figure 16 depicts the GOF for the regression-derived response of the ARMA model for the two acceleration sensors in the vertical direction and under different damage states versus the time of the test. As can be seen in Figure 16, the GOF is reduced as the deterioration progressed, which is in good agreement with the frequency change observation from the two last methods. Unlike the previous conventional methods which rely on the changes in the extracted modal features of the specimen, the proposed methodology relies on the ability of the fitted ARMA model to the pristine states’ vibration data, the reference ARMA model, for prediction of the response during a damaged condition. Another observation in Figure 16 is the slope of the fitted line to the GOF data. Based on the physics of the problem, it was expected that the sensor located close to the beam end would become more affected by the changes in the support region; therefore, a steeper fitted line was also expected.

**Figure 17:**
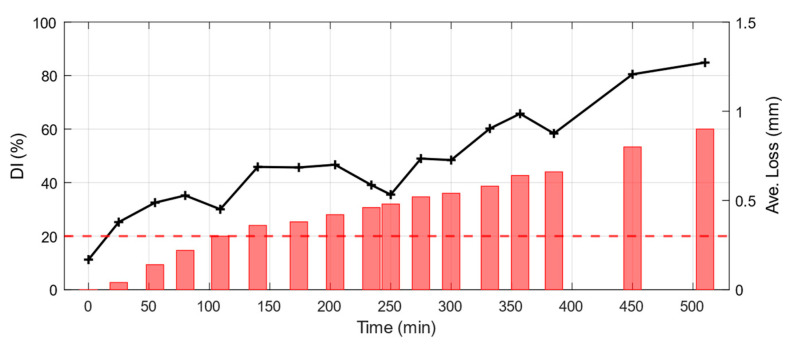
Damage index chart for the proposed methodology (black solid line with plus marker). Measured thickness reduction is shown in red bar on the right axis.

The results of the GOF for each sensor are then used to calculate the damage index for the specimen in hand. Figure 17 demonstrates the chart of the defined damage index for the test carried out. The red line indicates the threshold level which is defined empirically and based on the observations.

A comparison of damage index trends from three methods highlights differences in sensitivity to early-stage structural degradation. Conventional approaches struggle to distinguish between pristine and damaged conditions, showing only minor changes in *DI* (~1%) even in the case of advanced degradation. This limitation likely stems from their reliance on global metrics, i.e., natural frequency shift. On the other hand, the new DI, based on the GOF of predicted response from ARMA models in proposed method, shows a superior sensitivity to subtle material degradations. By capturing temporal dependencies and nonlinear dynamics, this approach detects small structural variations linked to damage. To quantitatively evaluate the relationship between the different damage indices from different methodologies with the level of applied damage, i.e., thickness loss, a linear regression analysis was performed. The Pearson correlation coefficient (*PCC*) was computed to measure the strength of the linear association between *DI* and thickness loss. The *PCC* value, ranging from −1 (indicating strong negative correlation) to 1 (strong positive correlation), indicates the degree to which *DI* can differentiate the degree of damage. A higher *PCC* suggests a stronger and clearer relationship. The *PCC* is calculated as follows:

(8) $$PCC=\frac{\sum (DI_{i}-\bar{DI})(TL_{i}-\bar{TL})}{\sqrt{\sum {\left(DI_{i}-\bar{DI}\right)}^{2}\sum {\left(TL_{i}-\bar{TL}\right)}^{2}}}$$

where $DI_{i}$ and $TL_{i}$ are the individual values of the damage index and thickness loss for each measurement, respectively, and $\bar{DI}$ and $\bar{TL}$ are their mean values. Table 5 shows the *PCC* values for different models. Although the *PCC* values for all methods show a strong correlation of *DI* with thickness loss, the highest obtained value corresponds to the proposed method.

## 4. Summary and Conclusions

This study explored the application of a novel ARMA-based regression approach for the early detection of material deterioration in steel structures. An accelerated electrochemical corrosion setup was employed to induce gradual controlled damage within a practical timeframe in a steel cantilever beam near its support. The progression of damage was verified through periodic thickness measurements, while the modal properties of the structure were assessed using acceleration data recorded via two attached sensors to the beam.

Three different methods were employed to analyze the vibration response: (I) the Covariance-Based Stochastic Subspace Identification (SSI-COV) algorithm, taken as reference modal analysis method and used for comparison; (II) a conventional ARMA model for time-series modeling; and (III) the proposed ARMA model regression, based on the goodness-of-fit metric. A damage index was introduced based on the goodness of fit between predicted and measured signals, effectively capturing material degradation trends.

A comparative study demonstrates that the proposed ARMA-based methodology offers superior resolution in detecting early-stage damage. The observed correlation between the average loss of thickness and the new defined damage index confirms the reliability of the proposed method. These findings underscore the potential of ARMA-based techniques for effective structural health monitoring of metallic structures, specifically in early-stage of damage evolution.

While the current study focuses on a steel material and a single type of corrosion, future work could extend the proposed methodologies to a broader range of materials and damage types. Additionally, a more detailed comparative analysis, particularly in terms of sensitivity and computational efficiency, remains an important avenue for further investigation. Preliminary findings suggest that the ARMA method is robust to parameter variations and computationally more suitable for edge deployment compared to the centralized SSI-COV method. However, a systematic sensitivity study and quantitative benchmarking of computational efficiency would provide a more comprehensive evaluation. Future studies could explore these aspects further to give a clearer understanding of the trade-offs between methods.

## Figures and Tables

**Table 1:** Simulated modal characteristics of the specimen.

Mode	Mode Description	Freq (Hz)
1	1st flexural	1.62
2	2nd flexural	18.10
3	1st Transversal	19.40
4	3rd flexural	55.65

**Table 2:** Measured thickness time history.

State	Time of Test (min)	Thickness @Zones of Measurement (mm)					Avg. Loss	Avg. *CR*
		P1	P2	P3	P4	P5	(mm)	(mm/h)
1	0	5.0	5.0	5.0	5.0	5.0	0.00	-
2	25	5.0	4.9	4.9	5.0	5.0	0.04	0.096
3	55	4.8	4.9	4.8	4.9	4.9	0.14	0.153
4	80	4.8	4.8	4.6	4.9	4.8	0.22	0.165
5	109	4.7	4.7	4.5	4.9	4.7	0.30	0.165
6	140	4.6	4.6	4.4	4.9	4.7	0.36	0.154
7	174	4.6	4.5	4.4	4.9	4.7	0.38	0.131
8	204	4.5	4.5	4.3	4.9	4.7	0.42	0.124
9	234	4.5	4.5	4.1	4.9	4.7	0.46	0.118
10	250	4.5	4.5	4.0	4.9	4.7	0.48	0.115
11	275	4.4	4.4	4.0	4.9	4.7	0.52	0.113
12	300	4.4	4.4	4.0	4.9	4.6	0.54	0.108
13	332	4.3	4.4	3.9	4.9	4.6	0.58	0.105
14	357	4.3	4.3	3.7	4.9	4.6	0.64	0.108
15	385	4.3	4.3	3.6	4.9	4.6	0.66	0.103
16	450	4.2	3.9	3.4	4.9	4.6	0.80	0.107
17	510	4.0	3.8	3.2	4.9	4.6	0.90	0.106

**Table 3:** AIC and *GOF* values for ARMA model selection.

Sensor 1 (Middle of Beam)				Sensor 2 (Free End of Beam)			
$\mathrm{AIC}\;(\times 10^{5}$)	GOF (%)	p	q	$\mathrm{AIC}\;(\times 10^{5}$)	GOF (%)	p	q
−6.667	83.89	26	36	−6.694	91.645	38	40
−6.667	83.87	28	42	−6.693	92.022	26	43
−6.665	84.05	26	41	−6.693	92.119	24	41
−6.664	82.74	20	41	−6.689	90.847	38	44
−6.663	83.62	34	36	−6.683	90.369	30	37

**Table 4:** Identified frequencies from SSI-COV and PSD of ARMA model.

State	SSI-COV		Conv. ARMA Model	
	Frequencies (Hz)		Frequencies (Hz)	
	Mode 1	Mode 2	Mode 1	Mode 2
1	1.6121	19.817	1.6125	19.8242
2	1.6104	19.896	1.6103	19.9356
3	1.6091	19.947	1.6103	20.0412
4	1.6093	19.841	1.6075	19.9901
5	1.6093	19.899	1.6072	19.7930
6	1.6083	19.849	1.6077	19.4601
7	1.6089	19.910	1.6077	20.0069
8	1.6098	19.880	1.6065	20.0982
9	1.6085	19.840	1.6073	19.8815
10	1.6073	19.912	1.6086	N.A
11	1.6061	19.860	1.6044	19.8438
12	1.6061	19.940	1.6041	N.A
13	1.6056	19.866	1.6043	19.8785
14	1.6026	19.833	1.6042	19.8258
15	1.6047	19.844	1.6042	19.8003
16	1.5999	19.791	1.6003	19.8011
17	1.5965	19.778	1.5974	19.6830

NA: Not Appeared in the analysis.

**Table 5:** Pearson correlation coefficient for different methods.

Method	PCC
SSI-COV	0.8991
Conv. ARMA	0.9374
Proposed Method	0.9394

## Data Availability

The data presented in this study are available upon reasonable request from the corresponding author.
